# The Economic Effects of Direct and Indirect Employee Involvement: Evidence From Corporate Social Responsibility Reports of Chinese Listed Companies

**DOI:** 10.3389/fpsyg.2021.762608

**Published:** 2021-10-15

**Authors:** Yongzhong Jiang, Xixi He, Yutao Zhu, Guosong Wu, Xinzhi Gao

**Affiliations:** ^1^College of Management Science, Chengdu University of Technology, Chengdu, China; ^2^The 29th Research Institute, China Electronics Technology Group Corporation, Chengdu, China; ^3^School of Economics and Management/“Two Mountains” Concept Research Institute, Huzhou University, Huzhou, China

**Keywords:** employee direct involvement, employee indirect involvement, corporate financial performance, the complementary effect, corporate social responsibility reports

## Abstract

Employee direct involvement and indirect involvement have been identified as essential forms of an enterprise’s democratic management in the digital economy. Research on the complementary effects of direct and indirect involvement is still in a blank state in China, which limits the external validity and accumulation of employee participation theory. The present study aimed to investigate the complementary effects of employee direct involvement and indirect involvement on the firm’s financial performance. Although previous research suggests that the influence of employee direct or indirect involvement on corporate financial performance has been examined separately, it is unclear whether the association between employee direct involvement and indirect involvement is complementary or conflictual. Based on strategic human resource management theory, we semantically encode 2,680 corporate social responsibility reports and the annual reports of 268 state-owned listed enterprises published from 2014 to 2018 *via* content analysis method, and the economic effects of employee direct involvement and indirect involvement were concurrently measured. We use configuration theory to explore the complementary effects between employee direct involvement and indirect involvement. Our results reveal that (1) employee involvement in Chinese enterprises was unbalanced, (2) both employee direct involvement and indirect involvement were positively related to enterprise’s financial performance, and (3) there is a complementary effect between the two forms of employee involvement. Theoretical and practical implications of these findings are discussed.

## Introduction

From 2000 to 2019, the number of labor dispute cases accepted and parties to such cases showed an upward trend: The number of labor dispute cases accepted reached 1,069,638 in 2019, for an increase of 691% over 2000 (National Bureau of Statistics in China, 2020). Notably, China’s problems with labor relations are serious. Consequently, employee involvement has great practical significance for the creation of a harmonious society. In recent years, with the development of a new generation of network information technology – cloud computing, big data, artificial intelligence, and blockchain – the digital economy is expanding at unprecedented speed, and it has led to major changes in technical characteristics and behavior on both the demand and supply side (Sutherland and Jarrahi, 2018). Corporate employees want to have more opportunities to express their views and be more involved in the democratic management of their enterprise (Breeze and Wiepking, 2020). In turn, employee involvement is conducive to communication between employees and the company and facilitates harmonious labor relations (Zhu et al., 2015). Therefore, it is crucial that we examine employee involvement in the digital economy.

Employee involvement is considered that employees involve in corporate management practices that affect their own work or working conditions to a certain extent in a specific way takes two forms: direct involvement and indirect involvement (Kandathil and Joseph, 2019). Employee direct involvement means that employees directly influence or control management practices associated with their work (Markey and Townsend, 2013), and employee indirect involvement refers to management practices that indirectly affect their working conditions through labor unions, employee congresses, and other institutions (Zeitoun and Pamini, 2021). Many scholars believe that employee direct involvement is conducive to improving corporate financial performance to a certain degree (Kim et al., 2010). In contrast, some studies have found that employee direct involvement does not promote corporate financial performance (Nayak and Sahoo, 2015). Overall, research on the impact of employee indirect involvement on corporate performance failed to reach a consistent conclusion (Cotton et al., 1988). Moreover, the literature on both employee direct involvement and indirect involvement is notably scant (Devaro, 2010).

Research on the complementary effects of direct and indirect involvement is still in a blank state in China, which limits the external validity and accumulation of employee participation theory. Employee direct involvement and indirect involvement usually occur at the same time in the course of business, and how different forms of employee involvement can be combined to affect corporate financial performance is at the forefront of Western employee involvement theory (Marchington, 2015). Some scholars propose that there should be complementary effects between different forms of employee involvement (Oehmichen et al., 2018), whereas other studies have found that there is no complementary effect, or even mutual interference, between the different forms of employee involvement (Teodorovicz et al., 2019). At present, few studies have used empirical methods to explore the possible interaction between employee direct and indirect involvement, and research conclusions differ (Zhu et al., 2018).

In the digital economy, what are the characteristics of the level of employee involvement in Chinese companies? Can employee involvement promote corporate financial performance? Are employee direct involvement and indirect involvement mutually reinforcing or do they interfere with each other? Research on employee involvement is mainly concentrated in the context of the Western free-market economic system; other institutional settings have rarely been examined in the literature (Peccei and Van De Voorde, 2019). To solve above problems, we adopt a strategic human resource management (SHRM) perspective, as well as human capital theory and resource-based theory, to examine the association between employee involvement and corporate financial performance. Additionally, we use configuration theory to explore the complementary effects between employee direct involvement and indirect involvement.

Accordingly, our study attempts to make several contributions to the literature. First, in accordance with the Guidelines for Compiling Chinese Corporate Social Responsibility Reports (CASS-CSR3.0), content analysis method is used to measure the two forms of employee involvement at the same time. Second, taking listed companies in China as the research object, we examine the economic effects of employee direct and indirect involvement, respectively, on a company’s financial performance. Third, for the first time in the context of the Chinese economic system, we also empirically test the complementary effects between employee direct and indirect involvement. The analysis proceeds as follows. The next section explains the theoretical basis of employee involvement’ effects on corporate performance and proposes the research hypotheses. Then, we present the research design, data sources, sample selection criteria, and variable definitions, and constructs the research model. Following that section, the results of the hypothesis tests are presented, and the last section discusses conclusions and makes suggestions for future research.

## Literature Review and Research Hypotheses

### Theoretical Issues

Strategic human resource management, which emerged in the 1980s, is a series of planned and SHRM planning and management actions taken by enterprises to achieve strategic goals (Lee et al., 2020). Early SHRM theory mainly emphasized situational theory, as well as the matching and contradictions between human resource practice and various strategies. Midterm SHRM theory mainly discussed the contribution of human capital and social capital to corporate strategy, and recent SHRM theory focuses on international human resource management in the digital economy (Wright and McMahan, 1992). Our brief literature review suggests that the association between human resource management practices and corporate financial performance is the research focus of SHRM (Guest, 2011). Therefore, SHRM provides an important theoretical basis for research on the association between employee involvement and corporate financial performance. Of these, resource-based theory, human capital theory, and configuration theory are the main theoretical foundations of SHRM. SHRM is a model for organizations to systematically plan and manage various human resource deployments and activities in order to achieve strategic objectives. It is an indispensable organic part of organizational strategy and one of the most important components of human resource management. Compared with traditional human resources management, SHRM is positioned to support the role and function of human resources management in the strategy of enterprises. That is a series of planned and strategic human resource deployment and management behaviors that enterprises can achieve their goals.

### Employee Direct Involvement and Corporate Performance

Resource-based theory holds that valuable, scarce, inimitable, and unique resources enable an enterprise to obtain a competitive advantage and emphasizes that resources are the decisive factor in organizational system and management (Wernerfelt, 1984; Barney et al., 2021). Enterprises succeed by acquiring and retaining scarce, valuable, and inimitable resources. Scholars have tried to explain how different corporate resources lead to better performance for companies (Chapman et al., 2018). For example, Xiao (2018) proposed that the scarcity of resources is characterized by hard to emulate. Although competitors can imitate a few human resource practices, it is difficult to duplicate the human resource management system formed by dominant companies through the organic combination of human resource practices. Wright et al. (2016) showed that even if competitors can completely copy the human resource system of an enterprise, such mimicry hard to produce the same effect because various enterprises have their own strategies and internal resources.

Drawing on resource-based theory, the SHRM school believes that human resource management practices can promote corporate performance (Barney, 2018). Under the pressure of fierce competition, most employees hope to have more say in their work through self-management. Every excellent employee is also eager for a fair evaluation and feedback on his work results and a scientific horse racing mechanism to stand out. Specifically, prior research showed that good human resource management practices will enable employees to form a positive work attitude, prompt them to have confidence in their future career development, and efficiently provide customers with excellent products and services. And a positive working atmosphere and better performance appraisal will lead to preferable employee output. This improves production efficiency to a certain extent, improves organizational output, and in turn affects corporate financial performance (Marin-Garcia and Bonavia, 2015). By setting goals together with employees, employees have a clear direction and correct ideas in their work, so that employees are willing to accept the constraints and guidance of work goals and generate great work motivation. Employee direct involvement is a human resource management practice often adopted by enterprises; this approach consists of individual-based involvement in the form of quality circles, work- and life-quality plan, self-management teams, employee symposia, high-level exchange meetings, factory affairs disclosure systems, employee satisfaction surveys, etc. The enterprise’s combination of these human resource management practices is a scarce resource that may have a positive effect on the company’s financial performance.

No consensus has been reached about the impact of employee direct involvement on corporate financial performance. Some experimental research shows that employee direct involvement has no direct impact on corporate financial performance. For instance, Rangus and Slavec (2016) found that the quality circle – an important form of employee direct involvement – did not make a beneficial contribution to corporate performance. In contrast, some empirical studies have found that employee direct involvement has a positive impact on corporate financial performance. For example, Salajegheh et al. (2015) used 132 employees of the Iranian Trade Bank as their sample and used analytic methods such as correlation analysis and regression analysis to empirically test whether a work- and life-quality plan can promote the improvement of corporate financial performance. In addition, corporate financial performance can be measured by corporate profitability, corporate operating capacity, corporate labor productivity, etc. (Ayuningtyas and Misnaniarti, 2016; Jones et al., 2016). Based on both resource-based theory and empirical results, it is possible that employee direct involvement is positively associated with corporate financial performance. Thus, we hypothesize:

H1: Employee direct involvement is positively related to corporate financial performance.

H1a: Employee direct involvement is positively related to corporate profitability.

H1b: Employee direct involvement is positively related to corporate operational capabilities.

H1c: Employee direct involvement is positively related to corporate labor productivity.

### Employee Indirect Involvement and Enterprise Performance

Human capital theory is another important theory of strategic human resource (Marginson, 2019). Human capital is a broad term that is used to describe the sum of economically valuable knowledge, skills, abilities, health, and other factors that exist in the human body, is acquired, and has great value to the organization (Becker, 1962). In other words, human capital can be thought of as an important strategic resource that is used to help companies gain substantial competitive advantages (Wright et al., 2016). Employee indirect involvement means that employees are involved in company affairs through their representatives, and representatives are usually elected by employee groups, for instance, trade unions, workers’ congresses, employee supervisory systems, employee committees, and joint advisory committees (Zeitoun and Pamini, 2021). According to previous literature, Brazen (2004) found that human capital investment has a significant role in promoting the return on investment (ROI) of enterprises and can bring economic value to the business. Skaggs and Youndt (2004) used 243 service companies as a sample and found that human capital has a significant positive impact on the return on equity and ROI of companies.

It is stated in human capital theory that companies need to invest in employees’ human capital through various human resource management practices – such as on-the-job training, the creation of corporate culture, and employee involvement – to improve corporate performance (Crook et al., 2011). In general, these institutional arrangements are conducive to enhancing workers’ collective bargaining power, seeking more human capital investment opportunities and benefits for employees, and accumulating human capital. Therefore, the indirect involvement of employees can increase the level of human capital investment in enterprises and promote the improvement of corporate performance. Prior research has shown that research on employee indirect involvement and corporate financial performance has not reached a consistent conclusion. For example, Laroche and Wechtler (2011) found that trade unions – an important form of indirect involvement – have not only failed to have a positive impact on enterprises, but have reduced enterprise productivity, product quality, and investment intensity. However, some empirical researchers have shown that employee indirect involvement has a positive impact on corporate financial performance (Shin et al., 2018). More importantly, China’s trade unions are different from those in the West. Western trade unions and employers usually have antagonisms, while the interests of China’s trade unions and units are consistent (John, 2010).

Taken together, it is rational to refer a positive correlation between employee indirect involvement and corporate financial performance. Therefore, this study proposes the following hypothesis:

H2: Employee indirect involvement is positively related to corporate financial performance.

H2a: Employee indirect involvement is positively related to corporate profitability.

H2b: Employee indirect involvement is positively related to corporate operational capabilities.

H2c: Employee indirect involvement is positively related to corporate labor productivity.

### The Complementary Effects of Employee Direct and Indirect Involvement

Configuration theory is one of the main perspectives on SHRM theory (Miles et al., 1978). Prior research has shown that the human resource management system is composed of multiple practical elements that interact to form a specific configuration and produce an overall synergistic effect. Different element-combination configuration methods – specifically, human resource configuration – will lead to differences in the overall effect of the system (Posthuma et al., 2013). As such, supporters of configuration theory believe that the combination of human resource practices has a significant association with the degree of employee-organization matching (Vekeman et al., 2019), and the human resource configuration can promote the knowledge management process of the enterprise and improve the enterprise’s performance (Singh, 2014).

Employee direct and indirect involvement have been widely adopted by modern enterprises as a form of human resource management practice that often exists in enterprise governance at the same time. According to configuration theory, when an enterprise simultaneously implements both direct and indirect involvement of employees, their effective allocation can create a synergistic effect and facilitate realization of the enterprise’s business objectives (Kato, 2006). However, Sengupta (2008) stated that they have failed to confirm the synergy of direct and indirect employee involvement. On the contrary, some scholars have found a mutual promotion effect between employee direct involvement and indirect involvement (Joseph et al., 2015). Thus, it is plausible that configuration theory might account for the association between two forms of employee involvement and enterprise financial performance, and we propose the following hypothesis:

H3: Employee direct involvement and employee indirect involvement have complementary effects in promoting enterprise financial performance.

## Materials and Methods

### Sample and Procedure

Because employee direct involvement and indirect involvement belong to the enterprise’s internal management know-how, companies usually do not publicly disclose this information; the exception is listed companies. In addition, China’s Company Law requires employee representatives on the supervisory boards of state-owned enterprises, but there are no mandatory regulations for non-state-owned enterprises. Therefore, we determined state-owned listed companies as the research target.

Data on employee direct involvement were collected in content analysis of listed companies’ corporate social responsibility reports. To ensure the continuity of data, 283 state-owned listed companies that have continuously disclosed corporate social responsibility reports on the Shanghai Stock Exchange since 2014 were selected as sample companies. After deleting companies with incomplete data, we obtained a valid sample of 268 listed companies. Data on employee indirect involvement are derived from the annual reports of listed companies. We conducted data extraction and content analysis of 1,340 annual reports and 1,340 corporate social responsibility reports of sample companies from 2014 to 2018. All data are derived from the Shanghai Stock Exchange and the China Stock Market & Accounting Research Database (CSMAR).

### Measures

#### Corporate Financial Performance

Profitability, operational capability, and productivity were designed to conduct a comprehensive and multi-angled empirical investigation of the financial performance of listed companies.

Profitability is expressed by the return on total assets – that is, the sum of total profits and financial expenses divided by average total assets. Operating capacity is expressed by the total asset turnover rate, which is the ratio of sales revenue to average total assets. Productivity is expressed by the logarithm of labor productivity (operating income per employee).

#### Employee Direct Involvement

We use content analysis to obtain measurement data on employee direct involvement by semantically coding social responsibility reports of sample enterprise. The specific coding method is as follows. If the listed company’s corporate social responsibility report does not disclose relevant information on employee direct involvement, it receives 1 point; if there is a keyword related to employee direct involvement, such as “suggestion box,” it receives 2 points; and if there are two keywords, it receives 3 points, and so on. If there are six or more keywords related specifically to employee direct involvement, it receives 7 points. Finally, we will obtain the scores of the variable DI, and the higher the score, the higher the degree of direct involvement of employees.

#### Employee Indirect Involvement

For employee indirect involvement (II), we obtain data on the level of employee indirect involvement in listed companies by counting the ratio of employee supervisors. Because some listed companies do not disclose information on this ratio, we use the following coding method: Companies that do not disclose this information receive 1 point; companies with a ratio greater than 0 but less than 0.3 receive 2 points; companies with a ratio equal to or greater than 0.3 and less than 0.4 receive3 points, and companies with a ratio greater than or equal to 0.4 receive 4 points.

#### Control Variables

In preliminary analyses, we evaluate whether other variables that may have an impact on the corporate financial performance should be controlled to rule out potential confounds. As a result, five variables that may affect financial performance are chosen be control variables: SIZE, LEVEL, AGE, LN (K/L), and LN (M/L). SIZE is the size of the company, measured by the natural logarithm of total assets at the end of the year; LEVEL is financial leverage, measured by the asset-liability ratio; AGE is the age of the company, which is 2018 minus the year is established; LN(K/L) is the natural logarithm of the capital-labor ratio – that is, the sum of net fixed assets and net intangible assets divided by the natural logarithm of the number of employees; Ln (M/L) is the natural logarithm of intermediate material input, which is measured by dividing the natural logarithm of operating costs minus salaries by the number of employees (Lopes and Godinho, 2019).

### Model Specification

Multivariable linear regression analysis is an appropriate tool to evaluate the links between the employee involvement and corporate financial performance variables. In particular, enterprise operating capacity is measured by the turnover rate of total assets, and profitability is measured by the rate of return on total assets.

The first sets of equations were estimated to test the influence of employee direct and indirect involvement on the company’s operating capacity and profitability and take the form:

$${\mathrm{AT}}_{i}=C+\beta_{1}{\mathrm{DI}}_{i}+\beta_{2}{\mathrm{CONTROL}}_{i}+\varepsilon_{i}$$ (1)

$${\mathrm{AT}}_{i}=C+\beta_{1}{\mathrm{II}}_{i}+\beta_{2}{\mathrm{CONTROL}}_{i}+\varepsilon_{i}$$ (2)

$${\mathrm{AT}}_{i}=C+\beta_{1}{\mathrm{DI}}_{i}+\beta_{2}{\mathrm{II}}_{i}+\beta_{3}{\mathrm{CONTROL}}_{i}+\varepsilon_{i}$$ (3)

$${\mathrm{ROA}}_{i}=C+\beta_{1}{\mathrm{DI}}_{i}+\beta_{2}{\mathrm{CONTROL}}_{i}+\varepsilon_{i}$$ (4)

$${\mathrm{ROA}}_{i}=C+\beta_{1}{\mathrm{II}}_{i}+\beta_{2}{\mathrm{CONTROL}}_{i}+\varepsilon_{i}$$ (5)

$${\mathrm{ROA}}_{i}=C+\beta_{1}{\mathrm{DI}}_{i}+\beta_{2}{\mathrm{II}}_{i}+\beta_{3}{\mathrm{CONTROL}}_{i}+\varepsilon_{i}$$ (6)

where for company i, AT_i_ is total asset turnover rate, ROA_i_ is return on total assets, C is a constant term, DI_i_ is employee direct involvement, II_i_ is employee indirect involvement, ε_i_ is an error term presumed to independent identical distribution, and CONTROL_i_ is a vector of company characteristics. The control variables include size of company, age of company, and asset-liability ratio. Using this extremely overall set of controls decreases the usual worry over omitted variable bias.

To test the impact of employee direct and indirect involvement on enterprise labor productivity and the complementary effects between the two, we augment Equation (7) with the Cobb–Douglas production function and estimate:


$$\mathrm{LN}{\left(Y/L\right)}_{i}=C+\beta_{1}{\mathrm{DI}}_{i}+\beta_{2}{\mathrm{II}}_{i}+\beta_{3}{\mathrm{DI}}_{i}*{\mathrm{II}}_{i}+\beta_{4}{\mathrm{CONTROL}}_{i}+\varepsilon_{i}$$ (7)

where for company i, LN(Y/L)_i_ is the natural logarithm of the labor productivity, CONTROL_i_ is a vector of company characteristics and other controls for company i, specifically, size of company, age of company, asset-liability ratio, the natural logarithm of the capital-labor ratio, and the natural logarithm of intermediate material input.

The β_3_ coefficients will preliminarily indicate the complementarity between employee direct involvement and indirect involvement.

## Results

### Preliminary Analysis

We used SPSS 23.0 statistical software to perform descriptive statistics on the data. As shown in Table 1, the average value of employee direct involvement (DI) in listed companies is only 2.47, and it indicates that the level of employee DI in state-owned listed companies is relatively low. In turn, it shows that China’s state-owned listed companies are not paying enough attention to DI of employees. In the process of performing content analysis on the listed companies’ corporate social responsibility reports, we found that listed companies not only have traditional employee DI methods – such as factory affairs disclosure systems, suggestion boxes, rationalization proposal, quality circles, employee symposia, and corporate intranet communication platforms – but also use innovative forms of employee DI, such as work- and life-quality plans, independent management teams, high-level exchange meetings, general manager reception days, employee satisfaction surveys, and new media communication platforms, such as WeChat and Weibo. However, the DI score for many enterprises is low, indicating that the degree of employee DI in enterprise management decision-making is not high. The variance of employee DI is 3.483, which reflects the imbalance in the level of employee DI in management in listed companies.

**Table 1:** Descriptive statistics and correlations (*N*=1,340).

S. No.	Variable	Mean	SD	1	2	3	4	5	6	7	8	9	10
1.	DI	2.47	1.866										
2.	II	2.7858	1.17464	−0.026									
3.	LN(Y/L)	14.1634	0.88655	0.095^**^	0.053								
4.	AT	0.6065	0.47888	0.030	0.040	0.307^**^	1						
5.	ROA	0.0286	0.05375	0.030	0.004	0.125^**^	0.123^**^	1					
6.	SIZE	23.8486	1.93284	0.152^**^	0.148^**^	0.360^**^	−0.162^**^	−0.021	1				
7.	AGE	17.1866	5.09458	0.051	−0.042	0.134^**^	−0.050	−0.063^*^	0.033	1			
8.	LEVEL	0.5647	0.20430	0.060^*^	0.068^*^	0.281^**^	−0.112^**^	−0.398^**^	0.539^**^	0.139^**^	1		
9.	LN(M/L)	14.0426	0.90549	0.092^**^	0.044	0.962^**^	0.389^**^	0.019	0.238^**^	0.118^**^	0.262^**^	1	
10.	LN(K/L)	14.1634	0.88655	0.060^*^	−0.041	0.203^**^	−0.182^**^	−0.041	0.135^**^	−0.027	−0.014	0.191^**^	1

*DI, direct involvement; II, indirect involvement; LN(Y/L), the logarithm of labor productivity; AT, total asset turnover rate; ROA, return on total assets; SIZE, size of the company; AGE, age of the company; LEVEL, financial leverage; LN(M/L), the natural logarithm of intermediate material input; LN(K/L), the natural logarithm of the capital-labor ratio*.

* *p<0.05 and*

** *p<0.01*.

The average value of employee indirect involvement (II) is 2.79, and it shows that state-owned listed companies have complied with the provisions of the Company Law and have a sufficient number of employee supervisors on the board of supervisors, which meets the basic requirements for employees to participate in management in terms of organizational form.

However, there are also a few companies that have not appointed employee supervisors or have not disclosed relevant information, which is contrary to national laws and regulations. The variance of employee II is 1.380, which reflects relatively balanced employee II at the management level in listed companies.

Then, Table 1 shows the correlations for our factor constructs. It was found that employee direct involvement was significantly and positively correlated with labor productivity (*r*=0.095, *p*<0.01). Thus, Hypothesis 1c is initially supported. The maximum correlation coefficient between employee direct involvement and employee indirect involvement and each variable is 0.152. There is no high correlation, so the multicollinearity problem can be excluded.

### Granger Test

Granger causality test is used to test whether one group of time series is the cause of another group of time series. Assuming that neither is a Granger cause, for both hypotheses the Granger causality test will give the value of *F* and the probability *p* that is greater than that value. If value of *F* is large and value of *p* is less than 0.05, then the null hypothesis is rejected and one variable can be regarded as the Granger cause of the other variable. Conversely, if the null hypothesis is accepted, one variable is not the Granger cause of the other. The Granger causality test between employee direct involvement and employee indirect involvement (Table 2) found no significant Granger causality between employee direct and indirect involvement. Therefore, it can be further confirmed that there is no multicollinearity problem among important explanatory variables.

**Table 2:** Results of the Granger test with employee direct involvement and employee indirect involvement.

Original hypothesis	Number	*F*-Statistic	Probability
The direct involvement of employees is not the indirect involvement of employees in Granger	267	1.73666	0.1887
The indirect involvement of employees is not the direct involvement of employees in Granger	267	0.07939	0.7783

*p>0.05 means there is no significant Granger causality between employee direct and indirect involvement*.

### Hypothesis Testing

Table 3 summarizes the findings of the hypothesis testing. The regression results of Models 1 and 2 show that employee direct involvement (DI) has a significant positive impact (*β*=0.059, *p*<0.05) on the total asset turnover (AT). On the other hand, employee indirect involvement (II) turned out to have a positive impact on the total asset turnover (*β*=0.070, *p*<0.05). Hypotheses 1b and 2b are therefore supported. The positive impact of employee direct involvement on the total asset turnover means that the higher level of employee direct involvement, the stronger the total asset turnover rate of the company – that is, the better the operational capabilities of the company. In addition, we found that the higher the level of employee indirect involvement, the stronger the enterprise’s total asset turnover – that is, the better the company’s operational capabilities.

**Table 3:** Regression analysis results.

Variables	AT			ROA			LN(Y/L)
	Model1	Model 2	Model 3	Model 4	Model 5	Model 6	Model 7
DI	0.059^**^ (2.153)		0.062^**^ (2.285)	0.057^**^ (2.279)		0.058^**^ (2.328)	0.032^**^ (2.018)
II		0.070^**^ (2.561)	0.073^***^ (2.675)		0.038 (1.504)	0.040 (1.578)	0.021^**^ (1.965)
DI*II							−0.058^***^ (3.250)
LN(M/L)							0.936^***^ (138.500)
LN(K/L)							0.003 (0.499)
SIZE	−0.144^***^ (−4.447)	−0.144^***^ (−4.465)	−0.155^***^ (−4.760)				0.175^***^ (22.354)
AGE	−0.018 (−0.651)	−0.011 (−0.038)	−0.014 (−0.478)	0.011 (0.414)	0.016 (0.604)	0.014 (0.533)	0.034^***^ (5.058)
LEVEL	−0.036 (−1.111)	−0.038 (−1.187)	−0.036 (−1.112)	−0.404^***^ (−15.938)	−0.404 (−15.891)	−0.407^***^ (−16.023)	−0.062^***^ (−7.999)
∑YEAR	control	control	control	control	control	control	control
Constant	1.445^***^ (7.834)	1.389^***^ (7.532)	1.413^***^ (7.663)	0.079^***^ (11.444)	0.077^***^ (10.112)	0.073^***^ (9.474)	−0.649^***^ (−5.439)
Observations	1,340	1,340	1,340	1,340	1,340	1,340	1,340
*F*	6.870^***^	7.120^***^	6.929^***^	38.187^***^	37.686^***^	33.762^***^	1981.668^***^
Adj. *R*^2^	0.034	0.035	0.038	0.163	0.161	0.164	0.947

DI, direct involvement; II, indirect involvement; DI*II, the interactive effect of direct involvement and indirect involvement; LN(M/L), the natural logarithm of intermediate material input; LN(K/L), the natural logarithm of the capital-labor ratio; SIZE, size of the company; AGE, age of the company; LEVEL, financial leverage; AT, total asset turnover rate; ROA, return on total assets; LN(Y/L), the logarithm of labor productivity.

*The numbers in parentheses are t-test values*.

** *p<0.01 and*

*** *p<0.001*.

For Model 3, we add variable II based on the Model 1. Model 3 shows that the regression coefficients between employee DI and company’s total AT are significant (*β*=0.062, *p*<0.05), and the regression coefficients of employee II on company’s total AT are significant (*β*=0.073, *p*<0.001). This once again shows that both direct and indirect involvement are significantly positively correlated with the company’s operating capacity. Hypotheses 1b and 2b are confirmed.

The regression results of Models 4 and 5 show that employee direct involvement (DI) has a significant positive impact (*β*=0.057, *p*<0.05) on the company’s total return on assets (ROA). On the other hand, the regression coefficient between employee II and the company’s total ROA was 0.038, but it is not statistically significant. Hence, supporting Hypotheses 1a but Hypothesis 2a is not supported. The positive impact of employee direct involvement on the company’s total ROA indicates that the higher the degree of employee direct involvement, the higher the ROA of the enterprise and the stronger the profitability of the enterprise. In addition, Model 5 indicates that employee indirect involvement does not affect the company’s profitability.

Model 6 use both employee direct and indirect involvement to perform a regression on total ROA. The results indeed suggest that employee direct involvement has a significant positive impact on total ROA (*β*=0.058, *p*<0.05). In contrast to that, we found a positive but non-significant association between employee indirect involvement and total ROA. Hence, Hypothesis 1a is supported again.

After controlling for variable, such as SIZE, LEVEL, AGE, LN (K/L), and LN (M/L), a plausible interpretation of the positive and significant associations between the presence of employee DI and company labor productivity (*β*=0.032, *p*<0.05) is that employee direct involvement and company labor productivity are significantly positively related. Thus, Hypothesis 1c is supported. Furthermore, the results of model 6 in Table 3 show that employee II has an impact positive significant on labor productivity of the enterprise (*β*=0.021, *p*<0.05). Hypothesis 2c is supported.

**Figure 1:**
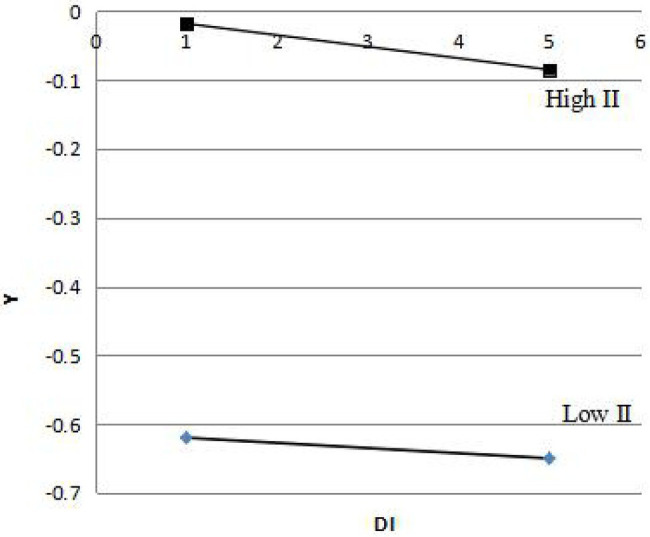
The interaction effect of employee indirect involvement on employee direct involvement. DI, direct involvement; II, direct involvement.

As we have observed, significant results are obtained for the interaction between employee direct involvement and indirect involvement (*β*=0.058, *p*<0.001). Following the recommendation of Pinsonneault and Kraemer (1993), the interaction effect was further tested. The high value of II was formed by adding the mean value of variable II to one standard deviation, and the low value of II was formed by subtracting the mean value of variable II from one standard deviation. When II was high, Y=−0.198DI+0.018, and when II was low, Y=−0.062DI−0.615. In line with above two regression equations, the DI regression coefficients have identical sign directions, and it indicates that employee indirect involvement has an enhanced interaction effect on employee direct involvement (Figure 1).

**Figure 2:**
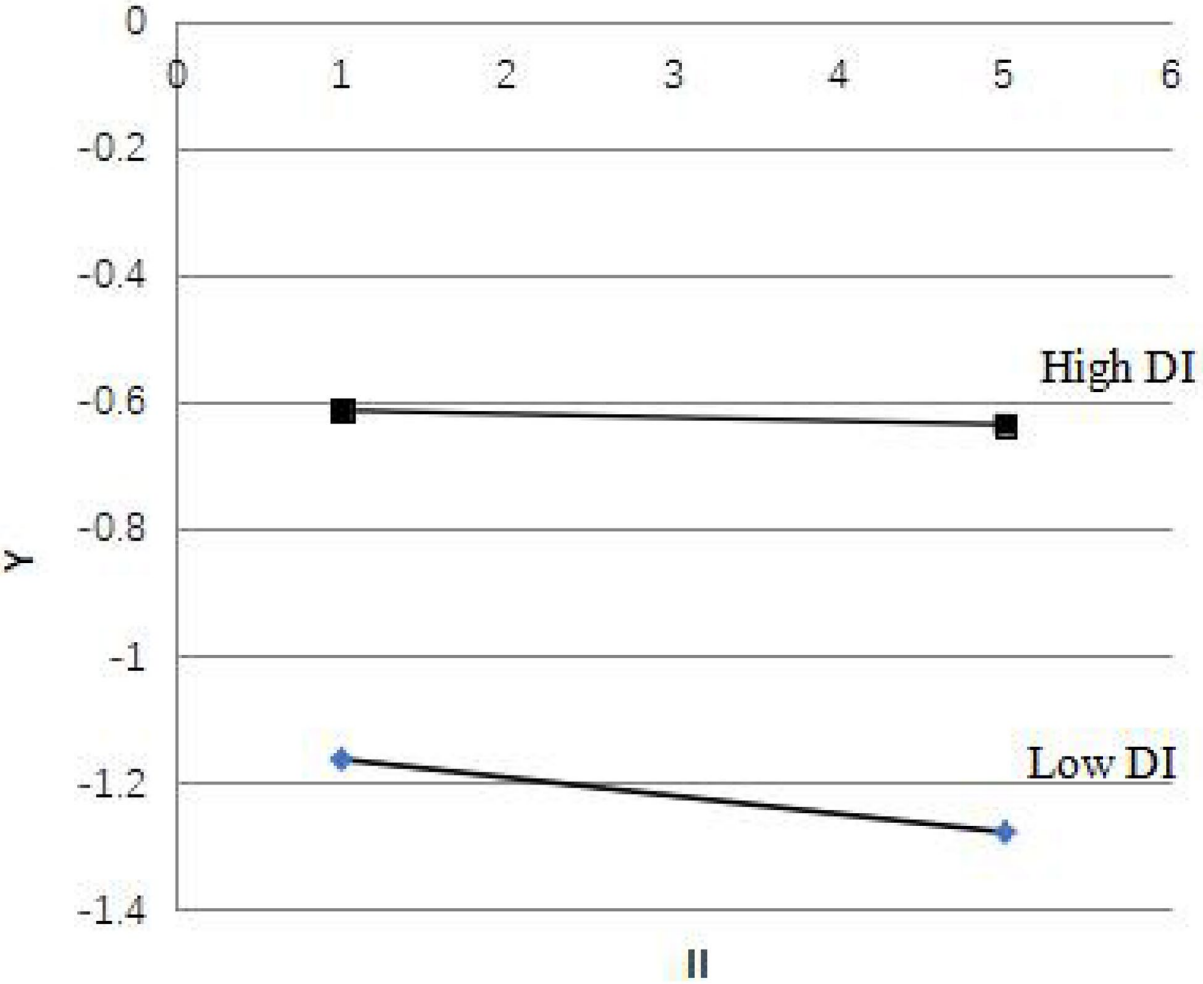
The interaction effect of employee direct involvement on employee indirect involvement. DI, direct involvement; II, direct involvement.

Similarly, when DI has a high value, Y=−0.231II−0.51, and when DI has a low value, Y=−0.014II−0.63. The II regression coefficients have identical sign directions, indicating that direct employee involvement also has an enhanced interaction effect on employee indirect involvement (Figure 2). Therefore, Hypothesis 3 was supported, which show that employee direct involvement and employee indirect involvement have complementary effects in promoting corporate financial performance.

To summarize, Hypothesis 1 is supported, Hypothesis 2 is mostly supported, and Hypothesis 3 is supported. The positive impact of employee direct involvement on the total asset turnover means that the higher level of employee direct involvement, the stronger the total asset turnover rate of the company – that is, the better the operational capabilities of the company. In addition, we found that the higher the level of employee indirect involvement, the stronger the enterprise’s total asset turnover – that is, the better the company’s operational capabilities. Employee direct involvement and employee indirect involvement have complementary effects in promoting corporate financial performance. This once again shows that both direct and indirect involvement are significantly positively correlated with the company’s operating capacity.

## Discussion

As scholars focused on network technology and the value of digital resource, it is hard not to notice the upsurge of employee involvement in the digital economy. Not only can technical breakthroughs offer convenience in the form of involvement, but they also have a clear effect on the financial performance of enterprises. In this study, we used state-owned listed companies that have continuously disclosed corporate social responsibility reports from 2014 to 2018 as our sample and simultaneously measured direct and indirect employee involvement for the first time and the effect of direct and indirect employee involvement on corporate financial performance. We found that there is an economic effect between direct employee involvement and indirect employee involvement.

### Theoretical Implications

Our findings in the current study have several important theoretical implications. First, this study extends previous research by providing support for resource-based theory, human capital theory, and configuration theory (Becker, 1962; Kato, 2006; Kozlenkova et al., 2014), which deepens our understanding of two forms of employee involvement. At present, China’s state-owned listed companies have generally established an employee supervisory system on the board of supervisors, and employees are indirectly involved in an organizational form that satisfies the requirements of China’s Company Law. However, listed companies do not attach enough importance to the direct involvement of employees, and the level of employee direct involvement in management is unbalanced. This reflects the main characteristics of the unbalanced and insufficient development of the level of employee involvement in Chinese enterprises in the digital economy.

Moreover, our study contributes to the enhanced theoretical understanding of the association between employee involvement and financial performance. In particular, our findings that employee direct involvement and indirect involvement are positively related to corporate performance provides support for resource-based theory and human capital theory (Sarason and Tegarden, 2003; Wright et al., 2016). Our results reveal that employee direct involvement and indirect involvement are significantly positively correlated with a company’s total asset turnover, return on total assets, and labor productivity. The results also show that the enterprise’s financial performance will be significantly improved when it attaches importance to employee direct involvement and indirect involvement, and employees’ voices are critical for the enterprise’s decision-making system. Our findings corroborate previous empirical studies that are consistent with the conclusions of Townsend et al. (2014). However, this study differs from previous studies, in that it simultaneously examines the impact of employee direct and indirect involvement on corporate financial performance.

Last but not the least, our study advances current knowledge by using a theoretical understanding of complementary effect. Specifically, our study demonstrates that employee indirect involvement has significant complementary effects in promoting corporate financial performance, which means that corporate financial performance improves when companies implement both employee direct involvement and indirect involvement practices. Our study adds the direct and indirect involvement interaction items (DI*II) of employees to the Cobb–Douglas production function model and finds that the direct and indirect involvement of employees has an enhanced interaction effect on corporate financial performance, this is consistent with the conclusion of Joseph et al. (2015). However, their research is based on a Western scenario, and the measurement method of employee involvement is different from our study. In general, we used a theoretical model that not only elaborates on why the two forms of employee involvement are related to corporate financial performance but also on their complementary effect.

### Managerial Implications

Our findings provide several important practical implications. Our findings demonstrate that employee direct involvement and indirect involvement are positively related to corporate financial performance and that both have enhanced complementary effects. Employees’ participation in enterprise management will not result from the situation of managers competing for power. They can only reasonably exercise the power given by the enterprise with the managers within their respective scope according to law, so that the enterprise can develop continuously. At the same time, employees can also improve their personal working ability. In encouraging employees to participate in all aspects, enterprises should proceed from reality and give reasonable play to the role of employee participation in enterprise management. In this light, we suggest that managers must attach great importance to employee involvement.

First and foremost, the state should incorporate employee involvement in legislation and implement top-level designs to respond to the increasing need for involvement by employees in China’s digital economy and strengthen the enforcement and supervision of laws and regulations, such as the Company Law, and gradually correct the unbalanced level of employee involvement by establishing an incentive and restraint mechanism whereby employees are involved in management.

Moreover, companies need to strictly abide by the provisions of the Company Law regarding the employee involvement system and implement their strategy for employee representation on the corporate board of directors and supervisors. Furthermore, companies must attach importance to employee involvement in system design and innovation. Companies must improve the disclosure system of corporate factory affairs and actively carry out high-level exchange meetings, symposia, democratic life meetings, and other activities to listen to employees. At the same time, companies must take full advantage of the technological dividends brought by big data and Internet technology and innovatively use the Internet, WeChat, Weibo, and other platforms to collect employee suggestions and conduct useful communication with employees. This is beneficial for breaking down the barriers of information imbalance and providing direct channels for employees to be involved in the company’s decision-making processes and management.

Finally, according to configuration theory, we found that employee involvement is not just a simple combination of participation practices (Kreiser et al., 2021). In order to maximize the effectiveness of employee involvement, companies must organically integrate the direct and indirect involvement practices of employees to derive the synergistic effect of 1+1>2.

### Limitations and Future Research Recommendations

The results of this research should be considered their limitations that may inspired future research directions. First, when measuring employee involvement, this study used publicly and objective data such as the annual reports and corporate social responsibility reports of listed companies. One limitation is that available sample is too small, and there are no available surveys of companies. Thus, future research may carry out large-scale corporate field surveys. In addition, this paper did not compare the impact of different types of enterprise employee involvement, and therefore, we recommend that future research conduct comparative studies on different types of enterprises. Finally, there may be some defects in the data acquisition of the research results, which may lead to a certain degree of autocorrelation or multicollinearity of the empirical results, which needs to be solved in the future research.

## Conclusion

To date, previous employee researches have been insufficient for understanding the complementary or conflictual association between employee direct involvement and indirect involvement. As such, our study aimed to resolve these deficiencies by employing a content analysis method and the semantic encoding of corporate social responsibility reports and the annual reports analyzing the economic effects of employee direct involvement and indirect involvement concurrently. Our results support resource-based theory, human capital theory, and configuration theory as a way to understand the nuanced association between employee direct involvement and indirect involvement. Our results also show the value of distinguishing the relationships between employee direct involvement and indirect involvement because they have significant complementary effects in promoting corporate financial performance. Our findings also suggest that organizational managers should implement sustainable organization strategy for employee representation on the corporate board of directors and supervisors and utilize the Internet technologies collecting employees’ voice to meet their growing psychological needs in digital economy.

## Data Availability Statement

The original contributions presented in the study are included in the article/supplementary material, further inquiries can be directed to the corresponding author.

## Author Contributions

YJ was responsible for the research design and conception, wrote the introduction and discussion sections, and revised accordingly. XH conducted theoretical analysis, proposed research hypotheses, and assisted in writing the research design. YZ collected and collated data, conducted data analysis, and assisted in results writing. GW collated the data and made the final revision of the paper. XG revised the paper for important intellectual content. All authors contributed to the paper and approved the submitted version.

## Funding

This work was supported by the National Social Science Foundation of China (grant no 16BZZ071), and the views expressed in this article are those of the authors and do not represent the foundation.

## Conflict of Interest

YZ is employed by China Electronics Technology Group Corporation.The remaining authors declare that the research was conducted in the absence of any commercial or financial relationships that could be construed as a potential conflict of interest.

## Publisher’s Note

All claims expressed in this article are solely those of the authors and do not necessarily represent those of their affiliated organizations, or those of the publisher, the editors and the reviewers. Any product that may be evaluated in this article, or claim that may be made by its manufacturer, is not guaranteed or endorsed by the publisher.
